# Development of a Generic PCR Detection of 3-Acetyldeoxy-nivalenol-, 15-Acetyldeoxynivalenol- and Nivalenol-Chemotypes of *Fusarium graminearum* Clade

**DOI:** 10.3390/ijms9122495

**Published:** 2008-12-05

**Authors:** Jian-Hua Wang, He-Ping Li, Bo Qu, Jing-Bo Zhang, Tao Huang, Fang-Fang Chen, Yu-Cai Liao

**Affiliations:** 1 Molecular Biotechnology Laboratory of Triticeae Crops, Huazhong Agricultural University, Wuhan 430070, P.R. China. E-Mails: jianhuawang@webmail.hzau.edu.cn (J. W); hepingli@mail.hzau.edu.cn (H. L.); qubo@mail.hzau.edu.cn (B. Q); jingbozhang@webmail.hzau.edu.cn (J. Z.); hhttao@mail.hzau.edu.cn (T. H); chenfangfang@webmail.hzau.edu.cn (F. C); 2 College of Plant Science and Technology, Huazhong Agricultural University, Wuhan 430070, P.R. China

**Keywords:** *Fusarium graminearum* clade, trichothecene, mycotoxin chemotype, 3-AcDON, 15-AcDON, NIV

## Abstract

*Fusarium graminearum* clade pathogens cause Fusarium head blight (FHB) or scab of wheat and other small cereal grains, producing different kinds of trichothecene mycotoxins that are detrimental to human and domestic animals. Type B trichothecene mycotoxins such as deoxynivalenol, 3-acetyldeoxynivalenol (3-AcDON), 15-acetyldeoxynivalenol (15-AcDON) and nivalenol (NIV) are the principal *Fusarium* mycotoxins reported in China, as well as in other countries. A genomic polymerase chain reaction (PCR) to predict chemotypes was developed based on the structural gene sequences of *Tri13* genes involved in trichothecene mycotoxin biosynthesis pathways. A single pair of primers derived from the *Tri13* genes detected a 583 bp fragment from 15-AcDON-chemotypes, a 644 bp fragment from 3-AcDON-chemotypes and an 859 bp fragment from NIV-producing strains. *Fusarium* strains from China, Nepal, USA and Europe were identified by this method, revealing their mycotoxin chemotypes identical to that obtained by chemical analyses of HPLC or GC/MS and other PCR assays. The mycotoxin chemotype-specific fragments were amplified from a highly variable region located in *Tri13* genes with three deletions for 15-AcDON-chemotypes, two deletions for 3-AcDON-chemotypes and no deletion for NIV-producers. This PCR assay generated a single amplicon and thus should be more reliable than other PCR-based assays that showed the absence or presence of a PCR fragment since these assays may generate false-negative results. The results with strains from several different countries as well as from different hosts further indicated that this method should be globally applicable. This is a rapid, reliable and cost-effective method for the identification of type B trichothecene mycotoxin chemotypes in *Fusarium* species and food safety controls.

## 1. Introduction

Fusarium head blight (FHB) or scab of wheat and other small cereal grains caused by *Fusarium graminearum* clade pathogens is an economically devastating disease worldwide [1]. FHB occurs both in the field and during storage, producing mycotoxins in moldy corn and wheat that are toxic to human and domestic animals [2–6]. Type B trichothecenes (8-ketotrichothecenes) are the principal toxins produced by *F. graminearum* clade. Based on the chemical structures and the acetylation positions of different 8-ketotrichothecenes, three trichothecene mycotoxin chemotypes have been identified within the type B trichothecene-producing *F. graminearum* clade: (IA) deoxynivalenol and 3-acetyldeoxy-nivalenol (3-AcDON), (IB) deoxynivalenol and 15-acetyldeoxynivalenol (15-AcDON), and (II) nivalenol and 4-acetylnivalenol (4-AcNIV) [7–10]. These trichothecene mycotoxins are difficult to detect and thus pose a serious risk to human health. Development of a fast, generic detection for DON, NIV and their acetylated mycotoxins will facilitate the molecular biology study and analysis of those mycotoxins in cereal grains and the derived products for food and livestock to reduce mycotoxin load.

Molecular characterization of trichothecene mycotoxin biosynthesis pathways has revealed the mycotoxin gene clusters and their regulations [5, 6, 11–16]. The *Tri13* gene has been found to be the determinant for the DON-NIV switching in *Fusarium*, and the *Tri7* gene is responsible for acetylation of NIV to produce 4-AcNIV. NIV-producers carry functional *Tri7* and *Tri13* genes while nonfunctional copies of both genes are present in DON-producers [17–20]. Comparative analysis of *Tri13* gene sequences from different *Fusarium* fungi revealed that the deletion sequences within *Tri13* gene appear to be associated with acetylation position of DON-chemotypes in addition to their association with the DON-NIV switching. Based on these sequences we have designed a pair of primers that allows amplification of 3-AcDON-, 15-AcDON- and NIV-chemotype-specific fragments of different sizes by PCR. With this method all isolates of *F. graminearum* clade should produce a trichothecene mycotoxin chemotype-specific product, which makes the PCR-based mycotoxin assays more efficient and reliable.

To demonstrate the applicability and reliability of this generic PCR detection, a global collection of *F. graminearum* clade strains from China, Nepal, USA and different European countries were assayed with this method together with GC/MS analysis. The results indicated that three different mycotoxin chemotypes of *F. graminearum* clade pathogens can be identified efficiently with this pair of primers, suggesting that this generic PCR detection could be used in the identification of mycotoxin chemotypes and food safety controls.

## 2. Materials and Methods

### 2.1. Fusarium Strains

All the Chinese *Fusarium* strains were selected from a large collection of *Fusarium* strains described previously [21]. They were isolated by single-spore isolation from the diseased wheat spikes that were collected from the regions with a known history of FHB epidemics in China. All the foreign *Fusarium* strains from France, Germany, Italy, Nepal, UK, and USA were obtained from the fungal collection of the John Innes Centre, UK, that were kindly provided by Dr. Paul Nicholson [22]. In total 54 strains of *F. graminearum* clade were used in this study, and their known chemotypes and detailed information are listed in Table 1.

### 2.2. DNA Extraction

*Fusarium* strains were grown on sterile glass-membrane paper overlaying potato dextrose (PDA) at 25 °C for 5 days. The mycelium were harvested and ground to fine powder in the presence of liquid nitrogen. Total genomic DNA was extracted using the CTAB method as described by Nicholson *et al*. [23].

### 2.3. Primer Design

Primers were designed with the aid of the Primer Premier 5 Program (PREMIER Biosoft International, Canada). The *Tri13* gene sequences of eight *F. graminearum* clade strains, including three NIV-producers (88-1, accession no. AF336365; HKM136, accession no. AY057841; HKM215, accession no. AY057842), four 15-AcDON producers (HKM87, accession no. AY057844; HKM95, accession no. AY057843; H-11, accession no. AF336366; GZ3639 accession no. AF359361) and one 3-AcDON producer (F15, accession no. AB060689) were compared through multiple sequence alignments. This allowed designing one set of primers Tri13P1 (5′-CTCSACCGCATCGAAGASTCTC-3′) and Tri13P2 (5′-GAASGTCGCARGACCTTGTTTC-3′) that generate an 859 bp fragment from NIV-producing strains, a 644 bp fragment from 3-AcDON-producers, and a 583 bp fragment from 15-AcDON-producers, respectively.

### 2.4. PCR Amplification

PCR reactions were carried out in a volume of 25 μL containing 50 ng DNA template and PCR reagents used were as described by Li *et al*. [24]. A negative control omitting DNA template was used in every set of reactions. The thermal cycler (Mycycler, Bio-RAD, USA) conditions used were: 94 °C for 4 min, followed by 35 cycles of 94 °C for 1 min, 58 °C for 40 s, 72 °C for 40 s, then a final extension of 72 °C for 6 min. PCR products were separated by electrophoresis on 2 % agarose gels, stained with ethidium bromide and photographed under UV light in the Bio-Imaging system (Bio-RAD, USA).

### 2.5. GC/MS Analysis

The strains were cultured in Petri dishes on the surface of a cellophane membrane laid over the PDA medium. After growth for 4 days in 28 °C, the mycelium was collected and ground as described above for DNA isolation. The powder was dried in electric blast drying oven. Supercritical fluid extraction (SFE) was used to extract mycotoxins. Extraction were analyzed by gas chromatography/mass spectrometry (QP2010, Shimadzu, Japan) as previously described by Maciej *et al*. [25] with the following modifications. The analysis was performed with a programmed temperature from 120 °C hold for 1 min, then to 280 °C at 20 °C min^−1^, and the final temperature being held for 8 min. The helium flow rate was held constant at 1 mL/min. The following ions were used for trichothecene detection: DON, *m*/*z* 235 and 422; 3-AcDON, *m*/*z* 117 and 392; 15-AcDON, *m*/*z* 193 and 392. The first ion in each set was used for quantitative analysis.

## 3. Results and Discussion

**Figure 1.**
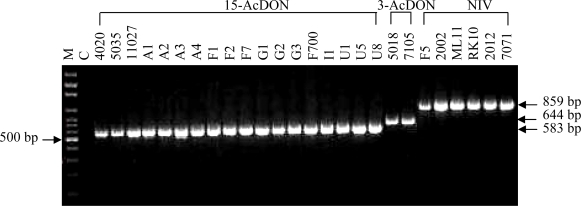
PCR amplification of 3-AcDON-, 15-AcDON- and NIV-chemotypes of *F. graminearum* clade strains. Lane M, 100-bp ladder marker; Lane C, negative control (omitting DNA template); Codes numbers above the panel correspond to the strain codes of *F. graminearum* clade in Table 1.

To investigate the reliability of the Tri13P1 and Tri13P2 primers for the identification of the 3-AcDON-, 15-AcDON- and NIV-chemotypes of *F. graminearum* clade, twenty six *F. graminearum* strains from various geographical origins were selected for the PCR assay. The mycotoxin chemotypes of these strains were determined either by HPLC, GC/MS or PCR. For instance, the strain 7015 from China was first determined to produce DON mycotoxin by HPLC [24] and is now identified as a 3-AcDON-producer by GC/MS (Table 1). The Chinese strain 5035 was identified as a 15-AcDON-producer by PCR assay [26] as well as GC/MS analysis (Table 1), while RK10 from Nepal produced NIV mycotoxins as revealed by both HPLC and PCR [24] (Table 1). PCR with Tri13P1 and Tri13P2 primers indeed showed that the 15-AcDON-chemotypes yielded a 583 bp fragment, a 644 bp fragment was generated from the 3-AcDON-chemotypes, and the NIV-chemotypes produced an 859 bp fragment. The three different chemotype-specific DNA fragments displayed a distinct profile on an agarose gel that could be easily determined by visual image under UV light (Figure 1).

With this method fifty-four strains from China, Europe, Nepal and USA were assayed and the results indicated that this pair of primers efficiently amplified a DNA fragment for all the strains with a chemotype-specificity. The results from this PCR assay were completely congruent with the previous chemical analyses and PCR identifications (Table 1) [22, 24, 26–28], indicating the high reliability for this generic PCR detection of three trichothecene mycotoxin chemotypes from *F. graminearum* clade strains.

**Figure 2.**
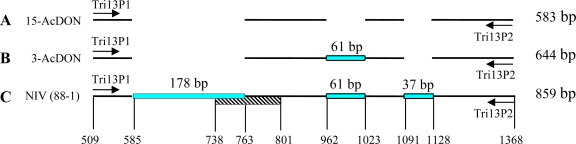
Diagrammatic presentations of *Tri13* genes are showing the gene structures of 3-AcDON-, 15-AcDON- and NIV-chemotype strains, and indicating the positions of primers designed for this study and the positions of nucleotides in the amplicon amplified by the primers in a NIV-chemotype.

The distinct DNA fragments from different chemotype-producing strains generated by this single pair of primers suggested that each chemotype contained a conserved structure within the *Tri13* gene sequences. NIV-producers carried an intact, functional *Tri13* gene with the region amplified by Tri13P1 and Tri13P2 primers, thus generating an 859 bp fragment (Figure 2-C). However, deletions were present in the *Tri13* gene sequences of DON-producing strains. 3-AcDON-chemotypes contained two deletions of 178 bp and 37 bp in length, respectively, that were located in the region spanned by the two primers Tri13P1 and Tri13P2, generating a 644 bp fragment (Figure 2-B). 15-AcDON-producers not only carried these two deletions but also had a third deletion of 61 bp in the region, yielding a 583 bp fragment (Figure 2-A). These structural characters ensure the efficient differentiation among the three chemotypes by this generic detection method.

The *Tri13* gene in the genome of NIV-producers encodes 3-acetyltrichothecene C-4 hydroxylase that plays an essential role for the addition of the C-4 oxygen to calonectrin [29]. The genome sequence of the *Tri13* gene contains 1802 bp with a unique intron of 63 bp between the positions 738 and 801 (Figure 2-C). The amplicon generated by the Tri13P1 and Tri13P2 primers includes the sequence from the positions 509 to 1368. The largest deletion of 178 bp fragment present in all DON-producers contains 153 bp of the first exon sequence (positions 585 to 737) and 25 bp of the intron (positions 738 to 763). The remaining two smaller deletions are located within the coding sequence of the *Tri13* gene. Sequence analyses showed these deletions in the DON-producing strains [30, 31]. The current study revealed that the numbers of the deletion within this region of the *Tri13* gene were apparently associated with the position of acetylation in DON-mycotoxin producers, which could be used as the molecular distinction between 3-AcDON- and 15-AcDON-chemotypes. Mechanisms involved in the creation of the chemotype-specific sequences in the *Tri13* genes during the evolution within *F. graminearum* clade remain to be investigated.

The generic PCR detection of 3-AcDON-, 15-AcDON- and NIV-chemotypes based on one amplicon should be more reliable than other PCR-based assays that showed the absence or presence of a PCR fragment since these assays may generate false-negative results. The results with strains from several different countries as well as from different hosts further indicated that this method should be globally applicable. This is a rapid, reliable and cost-effective method for the identification of three mycotoxin chemotypes in *Fusarium* species.

## 4. Conclusions

A single pair of primers based on the *Tri13* gene sequences of *F. graminearum* clade was designed that detected a chemotype-specific DNA fragment with different sizes from 3-AcDON-, 15-AcDON- and NIV-producers of *F. graminearum* clade strains. This PCR-based method was applied to assay the mycotoxin chemotypes of *Fusarium* strains from different countries and different hosts. The chemotypes revealed with this pair of primers were identical to that obtained by chemical analyses and other PCR-based assays. This generic PCR detection of the type B trichothecene mycotoxin chemotypes apparently appears to be more reliable and accurate than other PCR-based assays that may generate false-negative results based on the presence or absence of a DNA fragment. This is a reliable and cost-effective method for the identification of the trichothecene mycotoxin chemotypes in *F. graminearum* clade as well as in food and feed safety controls.

## Figures and Tables

**Table 1.** Origin, chemotype, host and PCR assay results of *Fusarium graminearum* clade strains examined in this study.

Strain code	Origin	Host	Chemotype	PCR assay results			
				Fragment size (bp)	NIV	3-Ac DON	15-Ac DON
2002	China	Wheat	NIV a	859	+	−	−
2012	China	Wheat	NIV a	859	+	−	−
3002	China	Wheat	15-AcDON a	583	−	−	+
4020	China	Wheat	15-AcDON b	583	−	−	+
5018	China	Wheat	3-AcDON c	644	−	+	−
5035	China	Wheat	15-AcDON d	583	−	−	+
5039	China	Wheat	3-AcDON a	644	−	+	−
5119	China	Wheat	15-AcDON a	583	−	−	+
5226	China	Wheat	n.t.	583	−	−	+
7105	China	Wheat	3-AcDON d	644	−	+	−
7047	China	Wheat	3-AcDON a	644	−	+	−
7071	China	Wheat	NIV a	859	+	−	−
7089	China	Wheat	3-AcDON a	644	−	+	−
11027	China	Wheat	15-AcDON a	583	−	−	+
12002	China	Wheat	NIV a	859	+	−	−
12003	China	Wheat	NIV a	859	+	−	−
13081	China	Wheat	n.t.	644	−	+	−
104	China	Wheat	n.t.	583	−	−	+
CH1-1	China	Wheat	n.t.	583	−	−	+
CH2-1	China	Wheat	n.t.	583	−	−	+
SH	China	Maize	n.t.	583	−	−	+
LY-11	China	Wheat	n.t.	583	−	−	+
YZ-2	China	Rice	n.t.	583	−	−	+
JYH	China	Maize	n.t.	583	−	−	+
F1	France	Wheat	15-AcDON c	583	−	−	+
F2	France	Wheat	15-AcDON c	583	−	−	+
F4	France	Wheat	15-AcDON c	583	−	−	+
F5	France	Wheat	n.t.	859	+	−	−
F6	France	Wheat	NIV c	859	+	−	−
F7	France	Wheat	15-AcDON c	583	−	−	+
D5	Germany	Wheat	DON e	583	−	−	+
G1	Germany	Wheat	15-AcDON c	583	−	−	+
G2	Germany	Wheat	15-AcDON c	583	−	−	+
G3	Germany	Wheat	15-AcDON c	583	−	−	+
F700	Germany	Wheat	15-AcDON b	583	−	−	+
G6	Germany	Wheat	15-AcDON c	583	−	−	+
I1	Italy	Wheat	15-AcDON c	583	−	−	+
I3	Italy	Wheat	15-AcDON c	583	−	−	+
ML11	Nepal	Maize	NIV e	859	+	−	−
RK10(HKM215)	Nepal	Rice	NIV e	859	+	−	−
N6 (MK6)	Nepal	Maize	NIV e	859	+	−	−
U1	UK	Wheat	15-AcDON c	583	−	−	+
U2	UK	Wheat	n.t.	859	+	−	−
U4	UK	Wheat	15-AcDON c	583	−	−	+
U5	UK	Wheat	15-AcDON c	583	−	−	+
U7	UK	Wheat	n.t.	583	−	−	+
U8	UK	Wheat	15-AcDON c	583	−	−	+
UK1	UK	Wheat	DON e	583	−	−	+
A1	USA	Wheat	15-AcDON c	583	−	−	+
A2	USA	Wheat	15-AcDON c	583	−	−	+
A3	USA	Wheat	15-AcDON c	583	−	−	+
A4	USA	Wheat	15-AcDON c	583	−	−	+
A5 (IL42)	USA	Wheat	15-AcDON c	583	−	−	+

+ Corresponding fragment amplified; −, no corresponding fragment amplified; n.t., Not tested.

a Mycotoxin chemotypes determined by PCR in Ref. [26];

b Mycotoxin chemotypes determined by HPLC in Ref. [24];

c Mycotoxin chemotypes determined by PCR in Ref. [27];

d Mycotoxin chemotypes determined by GC/MS in this study;

e Mycotoxin chemotypes determined by GC/MS in Ref. [22].
